# Magnetodielectric Properties of Ordered Microstructured Polydimethylsiloxane-Based Magnetorheological Elastomer with Fe_3_O_4_@rGO Nanoparticles

**DOI:** 10.3390/polym15040941

**Published:** 2023-02-14

**Authors:** Chunjie Zhang, Shaogang Liu, Fengmei Wei, Liqiang Dong, Dan Zhao, Yuqing Ou

**Affiliations:** 1 Department of Mechanical Design and Automation, College of Mechanical and Electrical Engineering, Harbin Engineering University, Harbin 150001, China; 2 Department of Software Engineering, College of Information Engineering, Harbin University, Harbin 150086, China; 3 Department of Software Engineering, College of Computer Science and Technology, Harbin Engineering University, Harbin 150001, China

**Keywords:** Fe_3_O_4_ nanoparticles, graphene oxide, ordered microstructured, magnetorheological elastomer, magnetodielectric effect

## Abstract

Magnetodielectric properties of prepared ordered microstructured polydimethylsiloxane-based magnetorheological elastomer with the Fe_3_O_4_@rGO (Fe_3_O_4_@rGO/PDMS-MRE) were investigated to expand the application of magnetorheological elastomer (MRE) in magnetic sensing fields by improving the magnetodielectric effect. Five types of Fe_3_O_4_@rGO electromagnetic biphasic composite particles were synthesized by the solvothermal method, and their characterization and magnetic properties were also tested. Microstructurally ordered Fe_3_O_4_@rGO/PDMS-MRE samples with different Fe_3_O_4_@rGO concentrations were obtained through the magnetic field orientation technique, an experimental platform for magnetodielectric properties was built, and the relative permittivity of the samples was tested under magnetic flux density from 0 to 500 mT. The results show when the ratio of modified Fe_3_O_4_ to GO reaches 10:1, the Fe_3_O_4_@rGO composite particles exhibit uniform distribution with a flaky structure and strong magnetic properties and have the best bonding effect of composite particles. The relative permittivity of Fe_3_O_4_@rGO/PDMS-MRE increases with the rise of Fe_3_O_4_@rGO concentration and applied magnetic flux density. The relative permittivity of Fe_3_O_4_@rGO/PDMS-MRE with Fe_3_O_4_@rGO concentration of 60 wt% reaches 12.934 under the action of 500 mT magnetic flux density, and the magnetodielectric effect is as high as 92.4%. A reasonable mechanism for improving the magnetodielectric effect of ordered microstructured Fe_3_O_4_@rGO/PDMS-MRE is proposed.

## 1. Introduction

Magnetorheological elastomer (MRE) is an emerging magneto-functional intelligent material that consists of a composite of magnetic particles and a polymer matrix [1,2]. The magnetic filler inside MRE undergoes displacement changes under the action of an external magnetic field, which is macroscopically manifested as a rapid, continuous, and reversible change in the mechanical and electrical properties of MRE controlled by the magnetic field, such as magnetorheological effect [3], magnetoresistance effect [4], and magnetic properties [5,6].

The magnetodielectric effect (MDE) is a phenomenon in which the capacitance or permittivity of a material changes under the action of the ambient magnetic field [7]. MDE has potential applications in novel devices, such as magnetic sensors, smart filters, data storage, and spin-charge converters [8,9], which has become another research hotspot after the MRE magnetoresistance effect [10,11]. With MRE as an intermediate dielectric for capacitors at the early stage, Bica’s team found that the capacitance value of MRE-based flat capacitors was significantly affected by the applied magnetic field, i.e., they had a certain MDE, which was due to the variation of the distance between the pole plates [12,13,14]. The team also investigated the effect of doping with different titanium dioxide (TiO_2_) concentrations on the MDE [15]. During the last five years, Bica’s team has prepared magnetorheological suspensions and hybrid magnetorheological elastomers (hMREs) and studied their MDE [16,17,18]. Semisalova et al. prepared MRE filled with various types of magnetic particles (Fe, NdFeB, Fe_3_O_4_) and compared the analysis of MRE-based capacitors applying a perpendicular magnetic field and parallel magnetic field cases [19]. The results showed that the MDE was more significant when the applied magnetic field was perpendicular to the capacitor pole plate, and it was also found that the magnetodielectric effect was related to the type, size, and concentration of the magnetic filler. The MDE was as high as 150% and 80% in the NdFeB and Fe powder-based MRE, respectively, which exhibited a strong magnetodielectric effect, but the MDE of the Fe_3_O_4_-based MRE was only 12~15%. Danil et al. presented a 3D model for simulating the magnetodielectric effect in MRE and compared the simulation results with the experimental data reported in the reference [19], which showed that the developed model described the effect qualitatively well [20]. Wang et al. investigated the fatigue dependence of the magnetodielectric effect of MRE, and they pointed out that for the “old” materials, the capacitance values tended to be higher for the same applied magnetic field intensity due to the destruction of their internal structure [21]. Belyaeva et al. studied hysteresis characteristics of MDE in magnetoactive materials [22].

Iron (II, III) oxide (Fe_3_O_4_) nanoparticles are oxide materials with both ferromagnetic and good conductive properties and exhibit superparamagnetic properties at room temperature, which can reduce the hysteresis error of magnetic sensors [23]. Polydimethylsiloxane (PDMS) has become a widely used matrix material in the sensing field because of its good flexibility, non-toxicity, low cost, easy processing, and chemical inertness [24]. Bi et al. studied the MDE of composites made by mixing Fe_3_O_4_ nanoparticles and PDMS with the methods of finite element and experiments, but the magnetodielectric effect was not significant [23,25]. As known to all, graphene, a new carbon-based conductor filler with superior electrical properties, can be added to polymers as a dielectric filler to enhance their dielectric properties [26]. Bica et al. prepared a cotton fabric-based MRE film using graphene nanoparticles as filler [27]. The results showed that the higher the graphene content, the higher the relative permittivity of MRE film. The team also tested the relative permittivity of MRE with different graphene contents at different magnetic field intensities based on the above MRE film and fitted the test results to obtain the approximate relationship between the relative permittivity of MRE and the magnetic field intensity at different graphene contents [28]. Additionally, the particle arrangement pattern has an effect on the relative permittivity of MRE, and Tsai et al. found that the relative permittivity of the material increased by 85% when the particles within the MRE were organized into a chain-like structure compared to that with a random distribution of particles [29].

The above study shows that the conventional magnetodielectric effect of Fe_3_O_4_-based MRE is poor, but the relative permittivity of MRE can be effectively improved by adding conductor fillers or changing the particle arrangement pattern, providing a new direction to improve the magnetodielectric effect of Fe_3_O_4_-based MRE. In addition, the formation of more microcapacitance in the polymer can enhance the charge storage capacity and improve the relative permittivity of the material. Moreover, Fe_3_O_4_@rGO composite particles by magnetic material Fe_3_O_4_ and reduced graphene oxide (rGO) have also received much attention at present because of their sensitive response to an external magnetic field and their wide application for the recoverable catalyst [30], drug carrier [31], and hydrogen evolution reaction [32].

Therefore, this paper developed an ordered microstructured polydimethylsiloxane-based magnetorheological elastomer (Fe_3_O_4_@rGO/PDMS-MRE) with magnetic field orientation technique based on the microstructure order and the microcapacitance principle. By synthesizing electromagnetic biphasic composite particles (Fe_3_O_4_@rGO), the filled particles can have both good magneto-control properties and conductivity, improve their dispersion in MRE, enhance the formation of microcapacitance inside MRE, and make MRE exhibit a strong magnetodielectric effect. This paper also explored the effect of Fe_3_O_4_@rGO concentration on the magnetodielectric properties of Fe_3_O_4_@rGO/PDMS-MRE and analyzed the mechanism of the magnetodielectric effect of MRE at the microscopic level as a reference for expanding its application in a magnetic sensing field.

## 2. Experimental

### 2.1. Materials

PDMS precursor and curing agent (Sylgard184, PDMS viscosity is 5500 mPa·s, and the mixture viscosity of PDMS and curing agent with the mass ratio of 10:1 is 4000 mPa·s) were provided by Dow Corning. The graphene oxide (GO) powder was purchased from Chengyi Education Science & Technology Co. Ltd., Wuxi, Jiangsu, China, with an average diameter of 20 μm. Fe_3_O_4_ nanoparticles with an average diameter of 20 nm were purchased from Shanghai Macklin Biochemical Co., Ltd., Shanghai, China. (3-aminopropyl) triethoxysilane (KH550, 99%, molecular weight is 221.4, Shanghai Yaohua Co., Ltd., Shanghai, China) was used as modifier. The ethanol (≥99.7%, Tianjin Tianli Chemical Reagent Co., Ltd., Tianjin, China) and the ethylene glycol (Tianjin Fuyu Fine Chemical Co., Ltd., Tianjin, China) were used as a dispersant. The CRCl8411 (Tianjin Chuangya Technology Co., Ltd., Tianjin, China) was used to insulate polyurethane coating. Additionally, all reagents were used without further purification. Distilled water was used in the experiment.

### 2.2. Synthesis of Fe_3_O_4_@rGO Electromagnetic Biphasic Composite Particles

**Figure 1:**
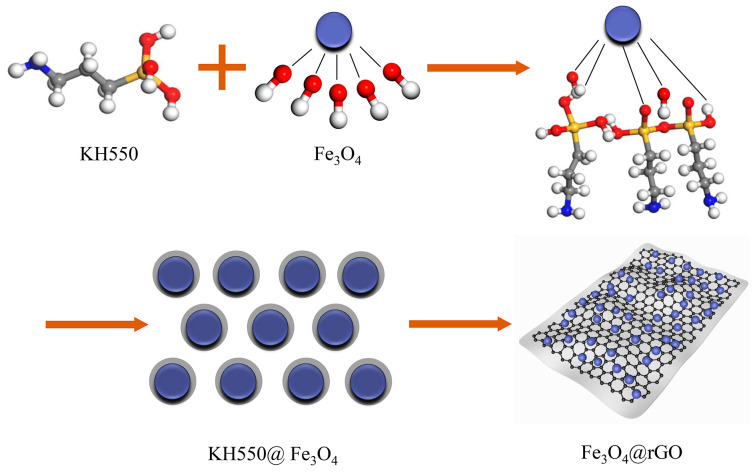
Schematic diagrams of the Fe_3_O_4_@rGO electromagnetic biphasic composite particles.

A total of 1.5 g of Fe_3_O_4_ nanoparticles were added to a mixed solution containing 12.5 mL of KH550, 45 mL of ethanol, and 5 mL of distilled water, and the products were mechanically stirred for 8 h and collected with a magnet. After gently washing with ethanol and distilled water in few amounts, the products were dried at 60 °C under vacuum, and modified Fe_3_O_4_ nanoparticles (i.e., KH550@Fe_3_O_4_) were obtained. Additionally, 90 mg of GO was put into 60 mL of ethylene glycol and ultrasonically shaken for 45 min to obtain the colloidal solution, and then the KH550@Fe_3_O_4_ particles were added to the colloidal solution and continued to be ultrasonically dispersed for 20 min. Subsequently, the colloidal solution of the mixture was added into 100 mL of PTFE reactor and held at 200 °C for 7 h. During this period, graphene oxide was reduced to graphene. After the reaction kettle was naturally cooled to room temperature, the product was transferred to a beaker, repeatedly washed with distilled water and ethanol, and finally freeze-dried for 24 h to obtain the black powder, namely Fe_3_O_4_@rGO electromagnetic biphasic composite particles (short for FG). By controlling the ratio of initial KH550@Fe_3_O_4_ and GO, five types of complexes ($m_{{\mathrm{Fe}}_{3}O_{4}}:m_{\mathrm{GO}}=1,\;5,\;10,\;15,\;20$ and the five different composite particles named FG-1, FG-5, FG-10, FG-15, FG-20) were prepared. The synthesis process of Fe_3_O_4_@rGO electromagnetic biphasic composite particles is shown in Figure 1.

### 2.3. Fabrication of Highly Ordered Fe_3_O_4_@rGO/PDMS-MRE

**Figure 2:**
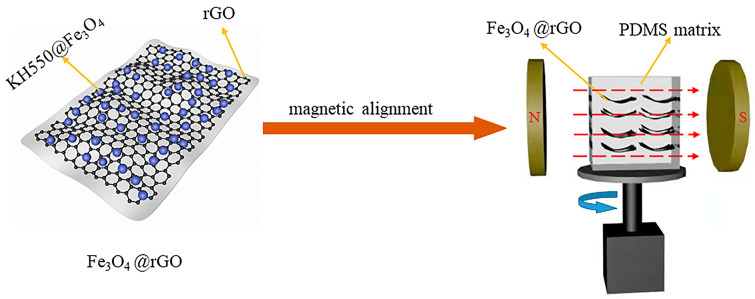
Schematic illustration of the fabrication process of highly ordered Fe3O4@rGO/PDMS-MRE.

Fe_3_O_4_@rGO was mixed with PDMS by solution mixing, and Fe_3_O_4_@rGO/PDMS-MRE with highly ordered microstructure was prepared by rotating magnetic field induction. The preparation process was as follows. First, Fe_3_O_4_@rGO was dispersed in appropriate amount of hexane and sonicated at room temperature for 15 min, and the required amount of PDMS was added and stirred uniformly for 45 min. Then, it was placed in a vacuum treatment at 100 °C environment for 1 h to evaporate the organic solvent inside. Finally, the curing agent (curing ratio 10:1) was added at room temperature. The mixture was further stirred vigorously for 30 min and de-bubbled in a vacuum drying oven at 60 °C, −0.08 MPa for 15 min, and then the mixed colloid was poured into a mold. At room temperature, the motor was turned on, and the sample was rotated at 30 r/min under magnetic flux density of 90 mT provided by NdFeB permanent magnets to form a rotating magnetic field so as to realize the orientation arrangement of Fe_3_O_4_@rGO particles parallel to the rotating plane of the external magnetic field. The sample was reversed at the same speed every 15 min, the magnetic field was oriented for 5 h, and the mold was removed and cured by heating at 90 °C for 2 h to obtain the microstructurally ordered Fe_3_O_4_@rGO/PDMS-MRE. The whole preparation process is schematically illustrated in Figure 2.

The specific samples prepared in this paper are shown in Table 1. A series of microstructurally ordered Fe_3_O_4_@rGO/PDMS-MRE samples (i.e., samples S_1_~S_10_ in Table 1) were prepared by the above preparation process. For the experimental comparison, rFe_3_O_4_@rGO/PDMS-MRE samples with random distribution of filled particles (i.e., sample S_11_ in Table 1), Fe_3_O_4_/PDMS-MRE (i.e., sample S_12_ in Table 1) and pure PDMS composites (i.e., sample S_13_ in Table 1) were also prepared. Where FG-i (i = 1, 5, 10, 15, 20) in Table 1 represents the mixing of KH550@Fe_3_O_4_ with GO at the mass ratio of i:1 in the preparation of Fe_3_O_4_@rGO composite particles.

### 2.4. Design of Magnetic Field Generator

In order to test the magnetodielectric properties of the prepared MRE samples, the experimental samples needed to be exposed to different magnetic fields during the testing process. In the previous literature, permanent magnets were used directly to adjust the gap between the magnets to control the magnetic flux density, resulting in a limited and unstable magnetic field adjustment range. Therefore, in this study, a new magnetic field generator has been designed to satisfy the need for MRE magnetodielectric properties testing. The magnetic field of this device was generated by an electromagnet, and the magnetic field of arbitrary intensity was obtained by adjusting the magnitude of the power supply current so as to ensure real-time adjustment of the intensity of the magnetic field applied to the axial direction of the MRE.

**Figure 3:**
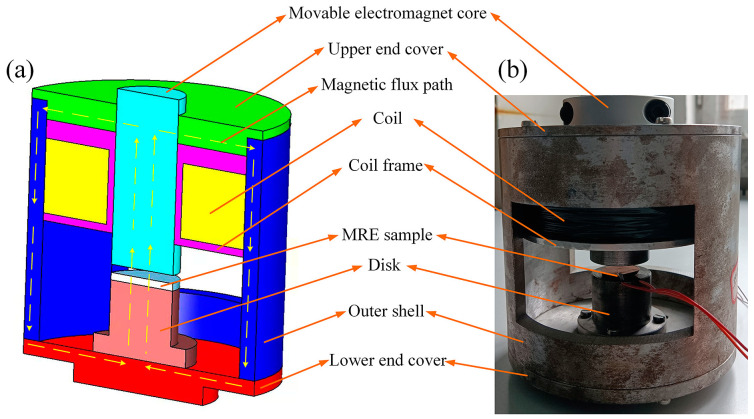
Magnetic field generator: (**a**) the 3D sectional model drawing, (**b**) the physical drawing.

The magnetic field generator mainly consists of a shell, upper and lower end covers, coil frame, coil, disk, and movable electromagnet core. In the magnetodielectric properties test, the MRE sample is placed between the upper and lower axes. Figure 3 shows the 3D sectional model drawing and the physical drawing of the magnetic field generator.

The magnetic field generator in this paper requires the magnetic flux density in the working area of MRE to reach 500 mT. To meet this requirement, we need to determine the structural parameters of the magnetic field generator, and the influence of the designed structural parameters on the magnetic circuit design needs to be considered. The structural design and magnetic circuit design are independent of each other and also affect each other, and the design of these two parts directly affects whether the magnetic field generator can achieve the expected results. Therefore, in the process of structural design, the structural parameters should be designed repeatedly, and the influencing factors should be considered comprehensively, and finally, the specific structural dimensions of the magnetic field generator are shown in Table 2.

**Figure 4:**
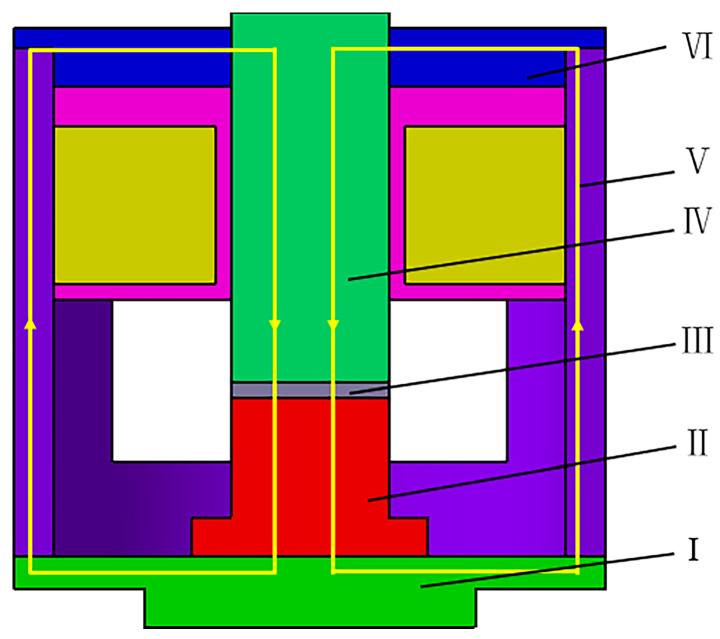
Schematic diagram of magnetic circuit structure.

The magnetic field is generated along the closed circuit when the electromagnetic coil is energized, as shown in Figure 4.

The specific calculation formula of the reluctance $R_{mi}$ is as follows:

(1) $$R_{mi}=\frac{l_{i}}{\mu_{0}\mu_{Di}S_{i}}$$

where $R_{mi}$ is the reluctance, $l_{i}$ is the average length of the *i*th magnetic circuit; $\mu_{0}$ is the air permeability (usually $\mu_{0}=4\Pi \times 10^{-7}\;H/m$); $\mu_{Di}$ is the relative permeability of the material; and $S_{i}$ is the average area of the *i*th magnetic circuit.

Based on the law of series magnetic circuit, the total reluctance of the entire circuit is derived as follows:

(2) $$R_{m}=\sum_{i=Ι}^{Ⅶ}R_{mi}=R_{mΙ}+R_{mⅡ}+R_{mⅢ}+R_{mⅣ}+R_{mⅤ}+R_{mⅥ}$$

The relationship among magnetic flux density, magnetic permeability, and magnetic flux:

(3) $$\left\{ \begin{array}{l} B=\mu H\\ \Phi =BS\\ \mu =\mu_{D}\mu_{0} \end{array}\right.$$

where B is the magnetic flux density, μ is the magnetic permeability, Φ is the magnetic flux, H is the magnetic field intensity, and S is the magnetic circuit area.

According to magnetic Ohm’s law:

(4) $$F_{m}=\Phi R_{m}=NI$$

where $F_{m}$ is the total magnetomotive force, $R_{m}$ is the total reluctance, N is the coil turns, and I is the current.

To ensure safety, the maximum current of the test is 2A, and the design parameters of the magnetic circuit determined with the above formula are shown in Table 3.

**Figure 5:**
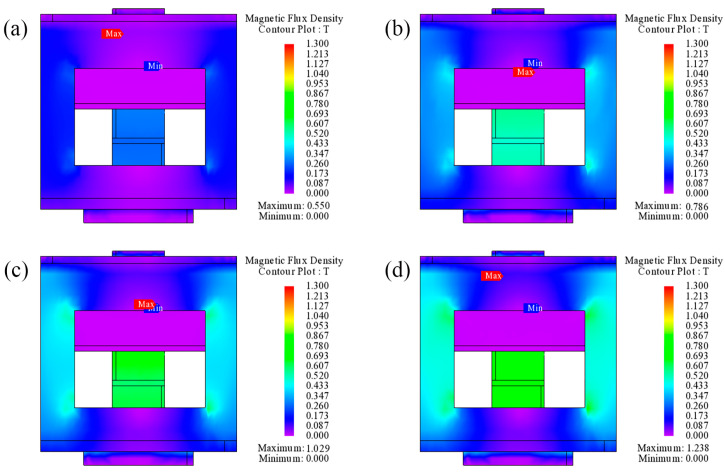
The simulation results of magnetic flux density of testing device under different currents. (**a**) distribution of magnetic field at the current of 0.5 A, (**b**) distribution of magnetic field at the current of 1 A, (**c**) distribution of magnetic field at the current of 1.5 A, and (**d**) distribution of magnetic field at the current of 2 A.

In this paper, JMAG-Designer magnetic field finite element analysis software was used to simulate the magnetic circuit of the magnetic field generator. Figure 5 shows the simulation results of magnetic flux density under different currents. As shown in Figure 5, under different currents, the distribution of magnetic flux density is proportional to the distance. The closer it is to the center of the shaft, the greater the magnetic flux density will be; otherwise, the smaller it is. The simulated magnetic flux density of MRE is 261 mT when the current is 0.5 A. It reaches 493 mT when the current is 1 A, 597 mT when the current is 1.5 A, and 693 mT when the current is 2 A. The magnetic flux density at the internal MRE structure can reach the expected target of 500 mT, and the design meets the requirement.

**Figure 6:**
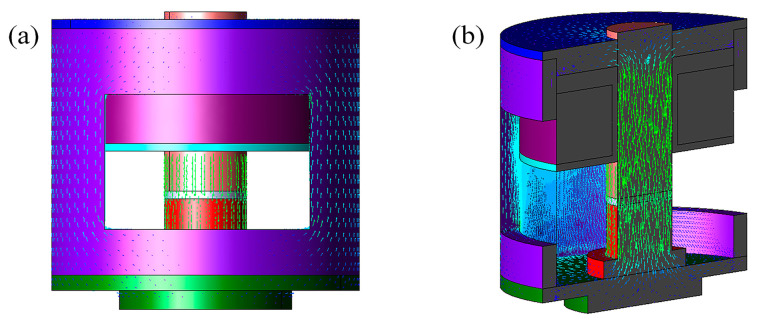
Distribution of magnetic field lines (**a**) the main view (**b**) the section view.

Figure 6 shows the distribution of magnetic field lines of the magnetic field generator, and it can be seen that the magnetic field lines pass through the movable electromagnet core, MRE, disk, lower-end covers, shell, and upper-end covers of the magnetic field generator in turn, and finally return to the movable electromagnet core, forming a closed loop. It is obvious from Figure 6b that the magnetic field lines acting on the MRE are uniformly distributed, and the environment in which the sample is placed is a uniform magnetic field.

**Figure 7:**
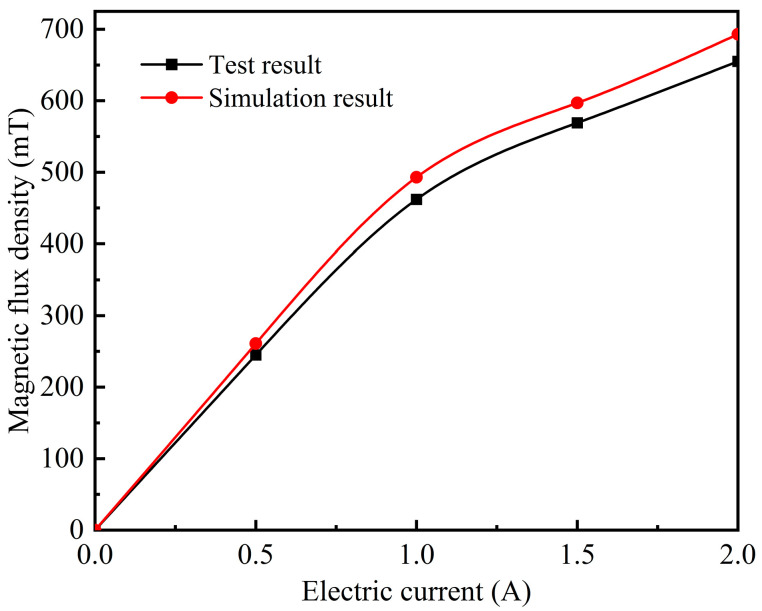
The relationship between the magnetic field intensity and the electric current.

In order to verify the reliability of magnetic field simulation results, the magnetic flux density near the MRE sample (i.e., <1 mm from the sample boundary) in the magnetic field generator was measured with a Tesla meter (Shanghai Huntoon HT20), and the measured result was compared with the finite element simulation result. As shown in Figure 7, the two trends are completely consistent, and the simulation result is slightly higher than the experimental result because there are phenomena such as magnetic loss or magnetic leakage in the actual experiment. The simulation data tally with the test data, confirming the feasibility and validity of the simulation.

### 2.5. Characterization and Property Testing

The XRD (Model TTR-III, Rigaku Corporation, Tokyo, Japan) was used for the phase analysis of Fe_3_O_4_@rGO composite particles with a scan speed of 7°/min and a scan range of 10°~80°. The FT-IR (Model Spectrum 100, PerkinElmer Corporation, Waltham, MS, USA) was used for the infrared testing of Fe_3_O_4_@rGO composite particles with a scanning range of 400~4000 cm^−1^. Transmission electron microscope (TEM) images and selected area electron diffraction (SAED) were recorded using a Talos F200S operating at 200 kV. The field emission scanning electron microscopy of high-tech thermal field type (Model SU5000, Hitachi High-Technologies Corporation, Tokyo, Japan) was used for the observation of the bonding effect of different types of Fe_3_O_4_@rGO composite particles and the cross-sections of Fe_3_O_4_@rGO/PDMS-MRE material, focusing on the arrangement of different Fe_3_O_4_@rGO concentrations inside Fe_3_O_4_@rGO/PDMS-MRE.

A vibrating sample magnetometer (Lake Shore 7407) was used to test the magnetic properties of the Fe_3_O_4_, the KH550@Fe_3_O_4,_ and the FG-10. The relative permittivity of samples in a zero magnetic field environment was measured by impedance analyzer (Model 6500B, Wayne Kerr Electronics), and the magnetodielectric properties of samples with different particle types and concentrations were measured by a magnetic field generator.

## 3. Results and Discussion

### 3.1. General Characterization

**Figure 8:**
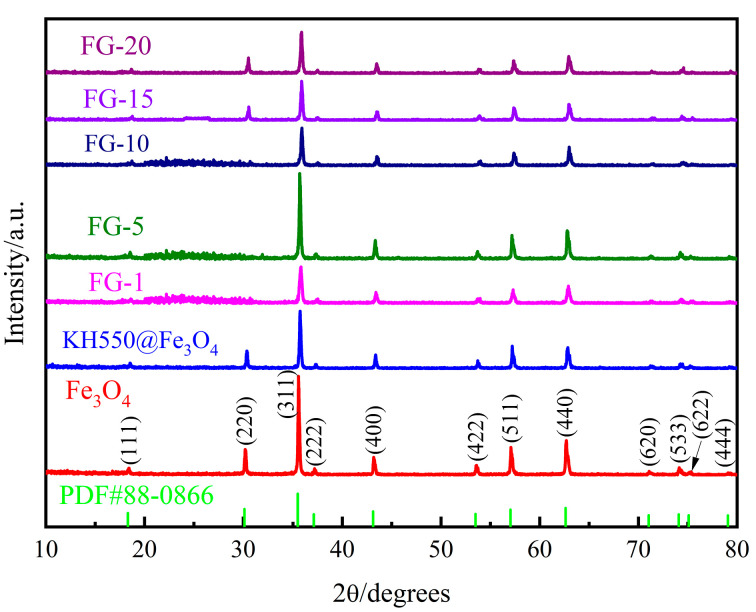
XRD spectra of Fe_3_O_4_, KH550@Fe_3_O_4,_ and Fe_3_O_4_@rGO with different types.

Figure 8 shows the XRD spectra of Fe_3_O_4_, KH550@Fe_3_O_4_, and different types of Fe_3_O_4_@rGO. The distinct diffraction peaks appear at 2θ = 18.3°, 30.1°, 35.5°, 37.1°, 43.1°, 53.5°, 57.0°, 62.6°, 71.0°, 74.1°, 75.1°, and 79.1°, corresponding to the crystalline planes of (111), (220), (311), (222), (400), (422), (511), (440), (620), (533), (622), and (444). This is consistent with the standard PDF card (PDF#88-0866) of Fe_3_O_4_ with an anti-spinel structure. According to the PDF card, the sample lattice structure is a cubic inverse spinel structure and belongs to the cubic system, Fd-3m (227), and the lattice constant is a = 8.3847Å. Apparently, the extremely similar curves of Fe_3_O_4_ and KH550@Fe_3_O_4_ prove that the modification of Fe_3_O_4_ by KH550 does not affect the Fe_3_O_4_ phase formation. Moreover, the characteristic broad peaks of Fe_3_O_4_@rGO between 20° and 30° correspond to the (002) crystal plane of rGO, revealing the successful synthesis of Fe_3_O_4_@rGO. The most obvious difference among the Fe_3_O_4_@rGO spectra of different ratios is that the broad peaks between 20° and 30° disappear and turn into flat lines when the Fe_3_O_4_ content rises, which is due to the high ratio and the high peaks of ferromagnetic materials that make it difficult to display the short, broad rGO peaks. The intensity of the broad peak belonging to rGO shown in Figure 8 gradually decreases with the decrease of rGO content, which is in accordance with the general rule of the composite particles prepared in the experiment. In addition, when the proportion of modified Fe_3_O_4_ exceeds 10, the characteristic diffraction peak intensity of (220) the crystal plane increases, the peak becomes sharp, and the crystallinity increases.

**Figure 9:**
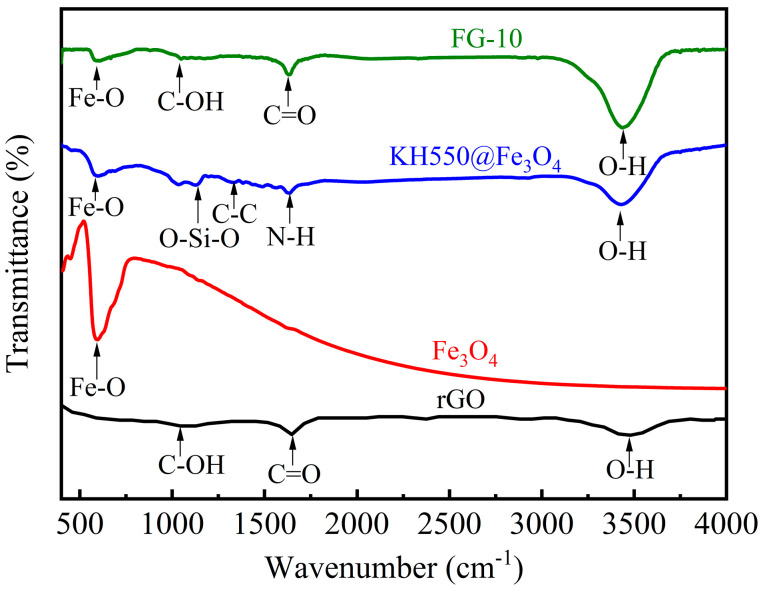
FT-IR spectra of rGO, Fe_3_O_4_, KH550@Fe_3_O_4_, and FG-10.

Figure 9 shows the FT-IR spectra of rGO, Fe_3_O_4_, KH550@Fe_3_O_4,_ and FG-10 composite particles. The overall rGO spectrum is relatively smooth, with the C-OH vibration peak at 1049 cm^−1^, the fine C=O peak occurring at 1642 cm^−1^, and the gentle O-H peak at 3450 cm^−1^, indicating that most of the oxygen-containing functional groups on the rGO surface have been removed and the reduction degree is high. In the spectrum of Fe_3_O_4_, the absorption peak at 595 cm^−1^ is attributed to the stretching vibration of the Fe-O bond. For KH550@Fe_3_O_4_, the stretching vibration peak at 589 cm^−1^ is also attributed to the Fe-O bond, but the peak intensity is lower than that of Fe_3_O_4_. The reason for the weaker peak intensity is that the relative content of the Fe-O bond decreases after KH550 is bonded to Fe_3_O_4_. The absorption peaks of stretching vibration at 1128 cm^−1^ are attributed to O-Si-O in KH550 [33], C-C single bond skeleton vibration at 1331 cm^−1^, N-H bond bending vibration at 1632 cm^−1^, and O-H bond stretching vibrations at 3431 cm^−1^, respectively. In the spectrum of FG-10, as a composite particle, it also has the characteristic peaks of a single substance, as shown by the absorption peaks representing the stretching vibrations of Fe-O, C-OH, C=O, and O-H bonds at 596 cm^−1^, 1049 cm^−1^, 1636 cm^−1^, and 3437 cm^−1^, respectively. Among them, the absorption peak of the stretching vibration at 1049 cm^−1^ is derived from the rGO sheets. The signal at 1636 cm^−1^ may be related to the C=O stretching vibrations of COOH groups situated at the edges of the rGO sheets. The peak intensity of the O-H bond becomes stronger because it has the characteristic peaks of both rGO and KH550@Fe_3_O_4_.

**Figure 10:**
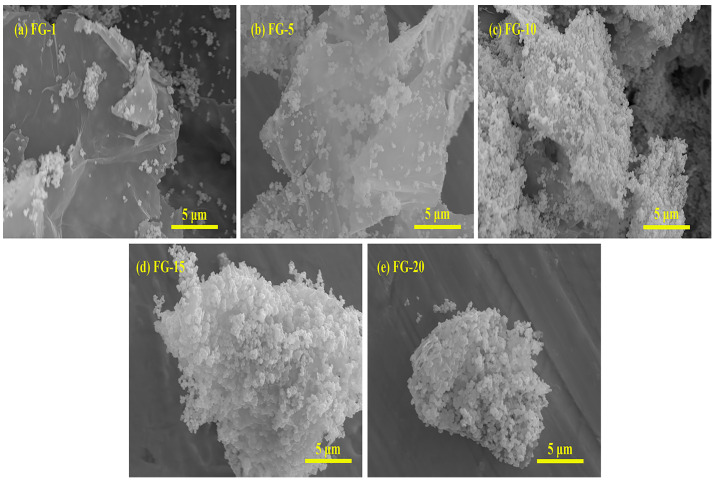
SEM images of Fe_3_O_4_@rGO composite particles with different types.

Figure 10 shows the SEM images of Fe_3_O_4_@rGO composite particles with different types. These pictures show that Fe_3_O_4_ nanoparticles are successfully compounded on the flexible graphene sheet. Additionally, the number of Fe_3_O_4_ particles gradually increases within the same field of view as the percentage of Fe_3_O_4_ rises, covering the graphene sheet more and more obviously. According to the SEM images of Figure 10a,b, the number of Fe_3_O_4_ loaded on the graphene sheets is low, and large uncovered areas appear. The Fe_3_O_4_ particles are uniformly distributed on the graphene surface in Figure 10c, and the composite particles have a flaky structure. The Fe_3_O_4_ stacking phenomenon starts to appear in Figure 10d. In Figure 10e, the Fe_3_O_4_ particles show an obvious agglomeration phenomenon, and the graphene’s flaky structure is no longer clear. SEM images show that the bonding effect of FG-10 composite nanoparticles works best.

**Figure 11:**
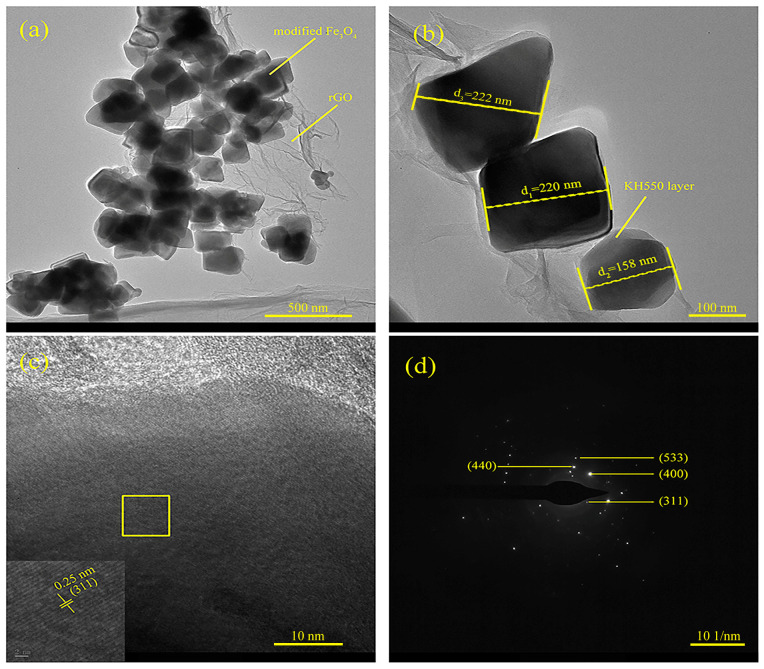
TEM images (**a**,**b**), HRTEM image (**c**), and SAED pattern (**d**) of FG-10 composite particles.

To further investigate the structural characteristics of Fe_3_O_4_@rGO composite particles, the morphological and crystallographic characteristics of FG-10 were observed by TEM, as shown in Figure 11. Figure 11a shows that the spherical Fe_3_O_4_ particles are uniformly fixed on the surface of rGO, which is consistent with the SEM observations. Figure 11b shows clearly the presence of a thin KH550 coating structure at the periphery of the Fe_3_O_4_ particles, which convincingly proves that KH550 successfully modified Fe_3_O_4_. It can also be found that the Fe_3_O_4_ particles coated by KH550 are still in the nanoscale, with an average particle size of 157 nm by measuring more than 30 particles in Figure 11a. According to the HRTEM image in Figure 11c, the lattice stripes with crystal plane spacing of 0.25 nm can be clearly observed, corresponding to the (311) Fe_3_O_4_ crystal planes. Additionally, the labeled diffraction rings can be guided to the (311), (400), (440), and (533) Fe_3_O_4_ crystal planes according to the selected area electron diffraction (SAED) photograph in Figure 11d, which is consistent with the previous XRD results.

**Figure 12:**
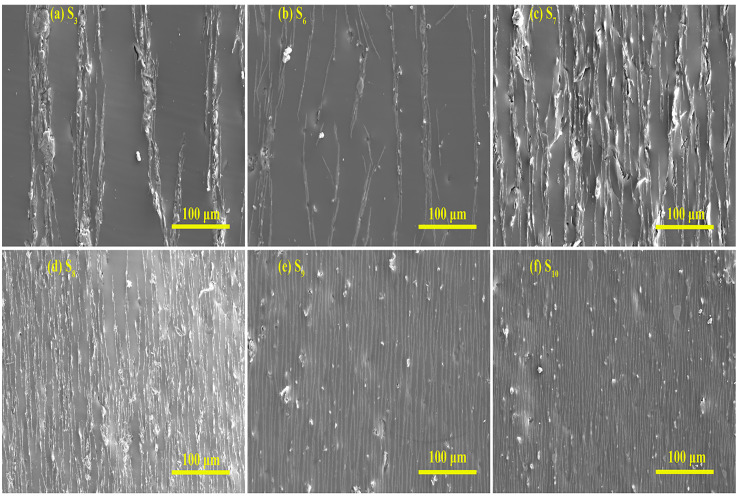
The longitudinal SEM images of microstructurally ordered Fe_3_O_4_@rGO/PDMS-MRE at different FG-10 particle concentrations.

Figure 12 shows the longitudinal SEM images of microstructurally ordered Fe_3_O_4_@rGO/PDMS-MRE at different FG-10 particle concentrations, and this group is the SEM images at a magnification of 300 times. The FG-10 particle concentrations are 10 wt%, 20 wt%, 30 wt%, 40 wt%, 50 wt%, and 60 wt%, corresponding to the sample numbers of S_3_, S_6_, S_7_, S_8_, S_9_, S_10_. It can be seen from the figures that the prepared samples form highly ordered microstructures internally, and the ordered microstructures become more obvious with the increase of FG-10 particle concentration. According to Figure 12a,b, it can be seen that the number of ordered microstructure columns is small, and each microstructure column is discontinuous when the concentration of FG-10 particles is low. When the concentration of FG-10 particles exceeds 40 wt%, it can be observed that the flaky FG-10 particles form uniform, fine, and ordered microstructures in the PDMS matrix with continuous microstructures per column, fully indicating that the Fe_3_O_4_@rGO particles have good orientation arrangement in the PDMS matrix.

**Figure 13:**
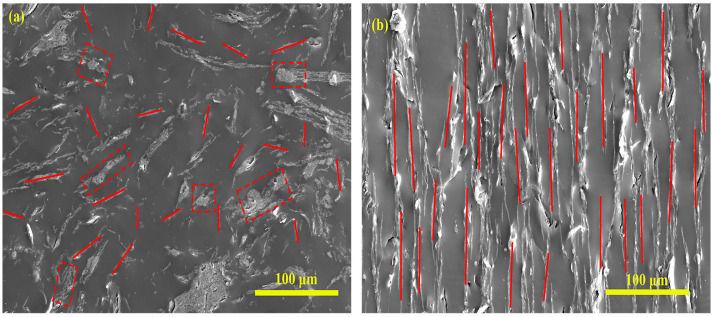
SEM images of Fe_3_O_4_@rGO/PDMS in longitudinal section (**a**) before the oriented arrangement (**b**) after the oriented arrangement.

The longitudinal section of Fe_3_O_4_@rGO/PDMS-MRE with 30 wt% concentration of FG-10 particles before and after the oriented arrangement was observed using SEM, as shown in Figure 13. Figure 13a shows the SEM image of the longitudinal cross-section of Fe_3_O_4_@rGO/PDMS-MRE with a random distribution of FG-10 particles, and it can be seen that a large number of nanosheets are disordered in the PDMS matrix. Figure 13b shows the longitudinal SEM image of microstructurally ordered Fe_3_O_4_@rGO/PDMS-MRE, and it is observed that the FG-10 particles are uniformly dispersed in PDMS and well oriented along the in-plane direction. It further indicates that the Fe_3_O_4_@rGO composite particles can achieve a high degree of directional alignment in the PDMS matrix under the magnetic field.

### 3.2. Magnetic Properties

**Figure 14:**
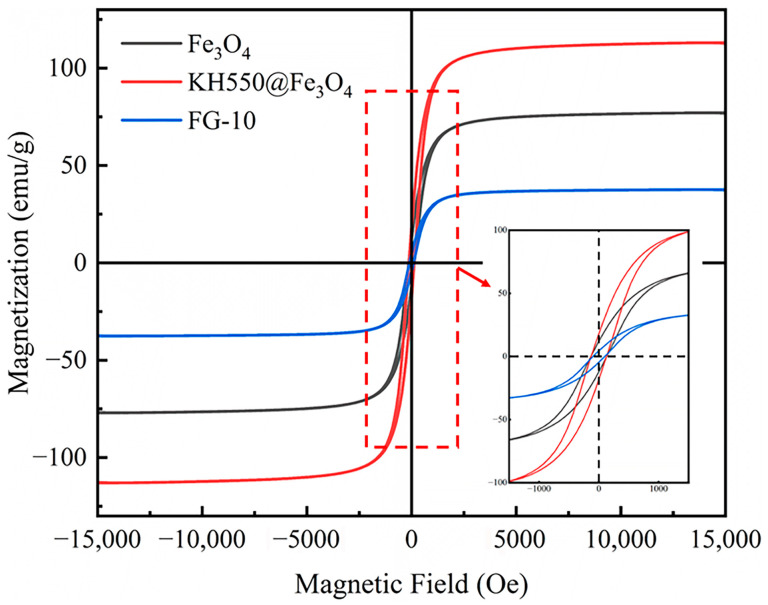
The magnetic hysteresis curves of Fe_3_O_4_, KH550@Fe_3_O_4_, and FG-10.

Figure 14 shows the magnetic hysteresis curves of Fe_3_O_4_, KH550@Fe_3_O_4_, and FG-10. The various magnetic property parameters of the three samples obtained from the magnetic hysteresis curves are shown in Table 4, mainly including saturation magnetization ($M_{s}$), remnant magnetization ($M_{r}$), and coercive force ($H_{c}$). According to the comparison between the Fe_3_O_4_ magnetic property parameters and KH550@Fe_3_O_4_ magnetic property parameters, the reason why the $M_{s}$ value of KH550@Fe_3_O_4_ increases is that the crystal particle size becomes larger after the modification of Fe_3_O_4_ by KH550, the ratio of its surface atoms decreases and the surface spin tilt phenomenon weakens, which in turn leads to a larger saturation magnetization value [34]. In addition, comparing the magnetic property parameters of FG-10 with that of KH550@Fe_3_O_4_, it can be seen that the introduction of graphene reduces the magnetic saturation strength of FG-10, but it still has superparamagnetic properties with a saturation magnetization strength of 42.36 emu/g, exhibiting low remanence properties.

### 3.3. Magnetodielectric Properties of Ordered Microstructured Fe_3_O_4_@rGO/PDMS-MRE

**Figure 15:**
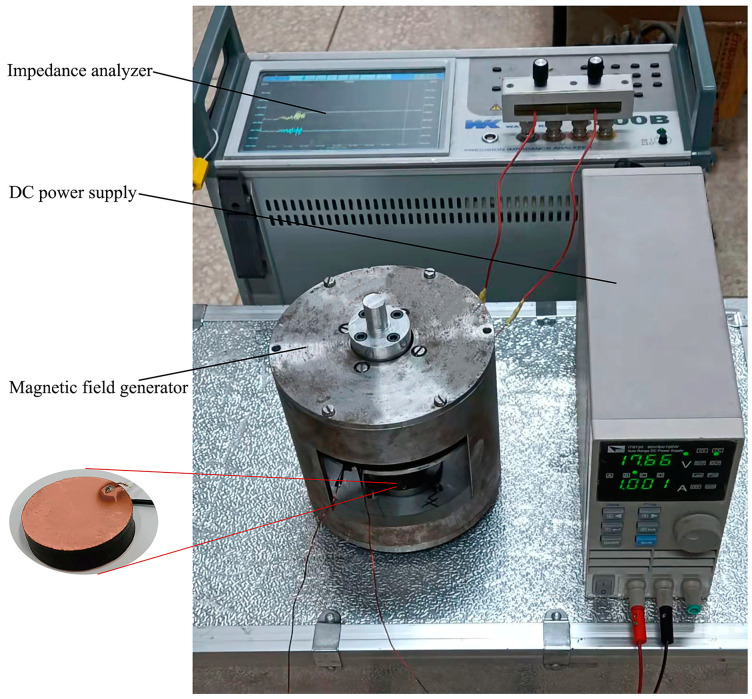
Test diagram of sample’s relative permittivity measurements in different magnetic environments.

In order to test the relative permittivity of ordered microstructure Fe_3_O_4_@rGO/PDMS-MRE under a magnetic field environment. The sample as a dielectric layer (diameter of 25 mm, thickness of 2 mm) was pasted with double conductive copper foil tapes on the upper and lower sides as the electrode material, and then the insulating polyurethane coating was uniformly sprayed on both sides of the upper and lower electrodes as the shielding layer. Copper wires were used to connect the sample to an impedance analyzer for magnetodielectric properties testing. At room temperature, the impedance analyzer combined with a magnetic field generator (capable of providing 0~500 mT magnetic field) is used to build the experimental test setup shown in Figure 15. It should be noted that the research shows that the magnetostrictive coefficient of MRE is roughly in the range of 10^−6^ to 10^−4^ [35], so the variation of the electrical plate spacing caused by the magnetic field was ignored during the test. During testing, the boundary effect of the electrode and the leads for measurement will generate additional capacitance, causing an error in the test data. In order to eliminate these test errors, the measured data were processed according to Equation (5).

(5) $$\varepsilon_{r}=\varepsilon_{r}{}^{\prime }-\varepsilon_{1}+1$$

where $\varepsilon_{r}$ is the true value of the relative permittivity of the sample; $\varepsilon_{r}{}^{\prime }$ is the measured value of the relative permittivity of the sample; $\varepsilon_{1}$ is the measured value when the dielectric material is air.

**Figure 16:**
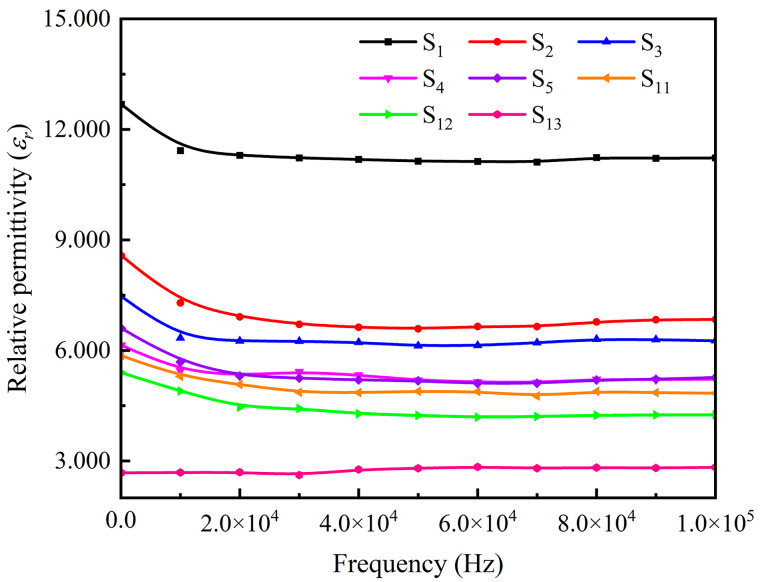
Frequency dependence of relative permittivity of pure PDMS and various MREs with 10 wt% filler content.

Figure 16 compares the relative permittivity of Fe_3_O_4_@rGO/PDMS-MRE with 10 wt% filler content with different ratios, Fe_3_O_4_/PDMS-MRE with 10 wt% filler content, and pure PDMS in the frequency range of 10^2^ to 10^5^ Hz under zero magnetic field. According to Figure 16, it can be seen that the relative permittivity of the materials decreases with the increase of the electric field frequency and tends to be stable with the change of the electric field frequency when the electric field frequency is greater than 30 kHz. The relative permittivity of pure PDMS is low, and its relative permittivity after stabilization is about 2.820. Comparing the S_i_ (i = 1,2,3,4,5) samples, the relative permittivity after stabilization of ordered microstructured Fe_3_O_4_@rGO/PDMS-MRE decreases with increasing the ratio of KH550@Fe_3_O_4_ to GO, which is attributed to the fact that the higher the ratio of fillers with the same concentration is, the less the relative number of composite particles is, and the less the number of microcapacitance can be formed in the matrix after orientation arrangement. In addition, comparing S_4_ with S_5_, we find that the relative permittivity of the material remains basically unchanged when the KH550@Fe_3_O_4_ to GO mass ratio exceeds 15:1, which may be attributed to the fact that with the increase of the ratio, the number of microcapacitances composite particles decreases, but increases the microcapacitance formed among Fe_3_O_4_ particles. The overall performance is that the relative permittivity of the composite exhibits basically unchanged overall, remaining at about 5.218. Comparing S_3_ with S_11_, it is found that the relative permittivity of Fe_3_O_4_@rGO/PDMS-MRE with an ordered microstructure of the same mass fraction of filler is larger than that of Fe_3_O_4_@rGO/PDMS-MRE with disordered microstructure. At the same time, the relative permittivity of the added composite particles materials is greater than that of Fe_3_O_4_/PDMS-MRE. It can be seen that the above analytical results fully demonstrate that the addition of ordered Fe_3_O_4_@rGO composite particles can effectively enhance the relative permittivity of MRE.

**Figure 17:**
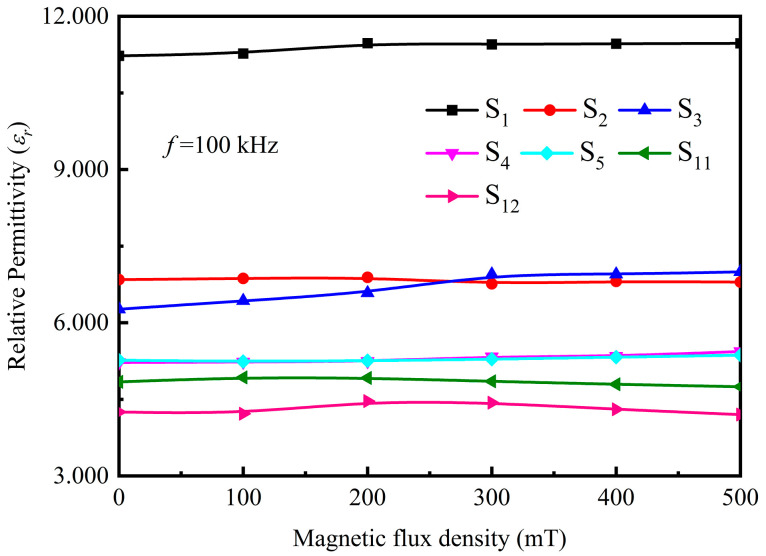
Relationship between relative permittivity of the samples and the external magnetic field.

The relationship between the relative permittivity and the applied magnetic field is obtained for samples with particle concentrations of 10 wt% at 100 kHz electric field frequency using the experimental setup in Figure 15, and the results are shown in Figure 17. During the test, the ambient magnetic field vector is parallel to the direction of the material excitation electric field, and the ambient magnetic field magnitude increases from 0 to 500 mT. It can be seen from Figure 17 that the relative permittivity of the samples increases with the enhancement of the magnetic flux density, showing the magnetodielectric effect. This is due to the decrease in the distance among the composite particles under the magnetic field, which causes a change in the inter-particle capacitance value. At the same time, the Fe_3_O_4_ particles have an orientation effect under the magnetic field, which changes the dipole moment of the electrons inside the particles and thus changes its own capacitance value, showing an increase in the relative permittivity of the material macroscopically. Among them, the microstructurally ordered Fe_3_O_4_@rGO/PDMS-MRE with the addition of FG-10 particles has the most obvious influence on the relative permittivity by the magnetic flux density, which increases from 6.263 to 6.995 by 11.7%, which may be attributed to the fact that the FG-10 composite particles with the best bonding effect and good magnetic properties are significantly affected by the applied magnetic field.

In addition, it is found that the relative permittivity of samples S_2_ and S_11_ decreases with increasing magnetic flux density, which is caused by the folding of the composite particles when receiving magnetic flux density. With the magnetic flux density of sample S_2_, due to the uneven distribution of Fe_3_O_4_ particles in the FG-5 composite particles, the position with more loaded Fe_3_O_4_ particles is subjected to a large magnetic force, while the position with less loaded Fe_3_O_4_ particles is subjected to a small magnetic force, leading to the folding in the flaky FG-5 composite particles. The stronger the magnetic flux density, the more obvious the folds in the flaky FG-5 composite particles. So the relative permittivity of sample S_2_ decreases with the increase of magnetic flux density. The FG-10 composite particles in sample S_11_ are randomly distributed, and there are many flaky FG-10 that are not parallel to the direction of the sample surface, and these FG-10 composite particles will be folded when receiving magnetic flux density. The stronger the magnetic flux density, the more obvious these FG-10 composite particles fold. So the relative permittivity of sample S_11_ also decreases with the increase of magnetic flux density.

**Figure 18:**
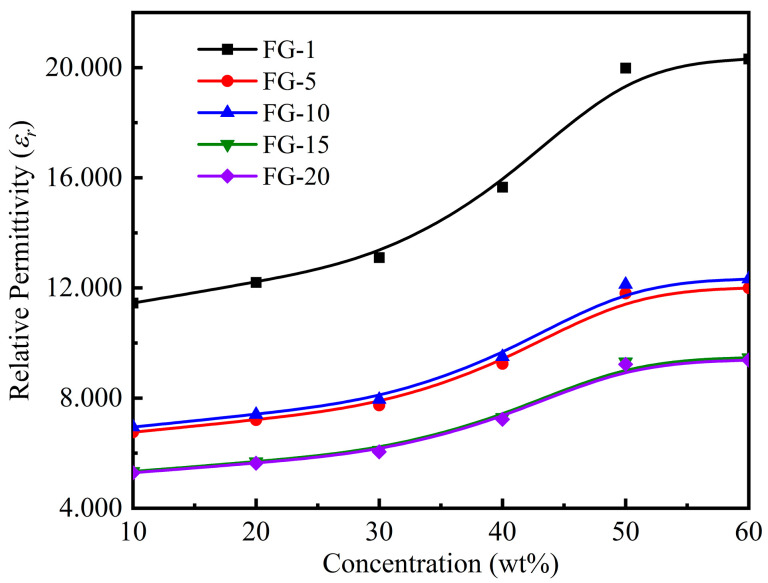
Relationship between relative permittivity of the samples and the particle concentrations.

Figure 18 shows the relationship between the relative permittivity and particle concentration of the microstructurally ordered Fe_3_O_4_@rGO/PDMS-MRE with different ratios of fillers added at a magnetic flux density of 300 mT. From Figure 18, it can be seen that the relative permittivity of MRE increases with the increase of particle concentration when the same proportion of filler is added, but when the particle concentration exceeds 50 wt%, the relative permittivity of MRE is basically unchanged, which may be due to the saturation of microcapacitance formed in the matrix by the composite particles, and the continued increase of particle concentration has little effect on the relative permittivity of the material. It can also be seen from the Figure that the relative permittivity of MRE decreases with the increase of filler ratio for the same particle concentration. This is attributed to the fact that for the same concentration of filler, the higher the ratio, the lower the relative number of composite particles and the fewer microcapacitances formed inside the matrix after the directional arrangement. Comparing the two curves with filler FG-15 and FG-20, it can be found that the relative permittivities of the materials are basically the same. This is due to the fact that a large amount of Fe_3_O_4_ accumulates on the rGO surface at a filler ratio of 20, which enhances the electron polarization and thus increases the relative permittivity of the materials. In addition, we also found an interesting phenomenon that the relative permittivity of MRE with filler FG-10 is higher than that of MRE with filler FG-5. This is due to the fact that with less and non-uniform Fe_3_O_4_ loaded on the surface of FG-5 particles and under the effect of a higher magnetic field, the flaky FG-5 particles are folded, which affects the formation of microcapacitance and thus decreases the relative permittivity of MRE with filler FG-5.

**Figure 19:**
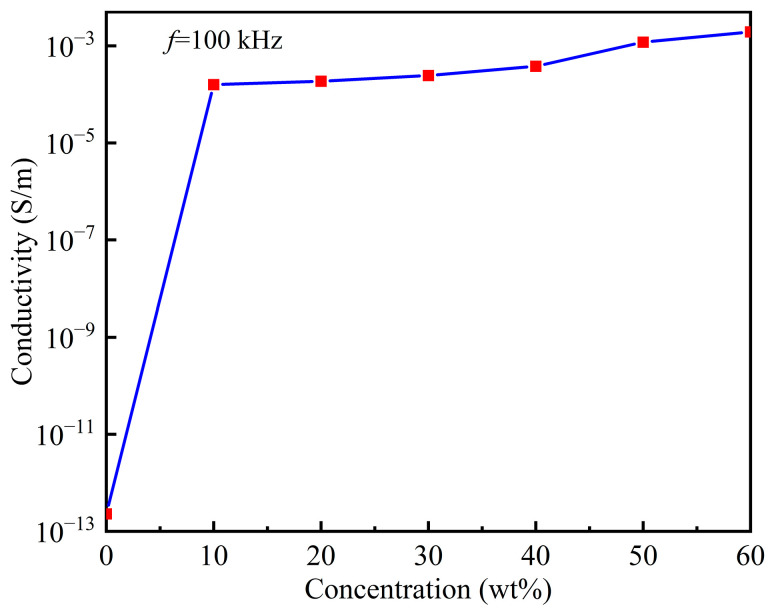
Relationship between conductivity of the samples and the particle concentrations.

To investigate the effect of particle concentration on the conductivity of the samples, we test the conductivity of the samples with different FG-10 particle concentrations at an electric field frequency of 100 kHz and a zero magnetic field, and the results are shown in Figure 19. The FG-10 concentrations are 0, 10 wt%, 20 wt%, 30 wt%, 40 wt%, 50 wt%, and 60 wt%, respectively. It can be seen from the Figure that the conductivity of pure PDMS is 10^−13^ S/m, and when the FG-10 concentration is increased by 10 wt%, the conductivity of the material increases steeply by 10^9^ times, indicating that the addition of FG-10 can fully improve the conductivity of the material. When the concentration of FG-10 is increased from 40 wt% to 50 wt%, the conductivity of the samples increases significantly by an order of magnitude, with the conductivity of the samples reaching 10^−3^ S/m. When the concentration of FG-10 is increased from 50 wt% to 60 wt%, the trend of increasing the conductivity of the materials becomes flat due to the near saturation of the polymerization ability between the particles. In addition, the trend of the sample conductivity with particle concentration is consistent with the variation of the particle spacing in the SEM images of the samples with different particle contents in Figure 12.

We defined the magnitude of the magnetodielectric effect by the following expression:

(6) $$MDE(\%)=\frac{\varepsilon_{H}-\varepsilon_{0}}{\varepsilon_{0}}$$

where $\varepsilon_{H}$ and $\varepsilon_{0}$ are the relative dielectric permittivity values of ordered microstructured Fe_3_O_4_@rGO/PDMS-MRE without magnetic field and when the magnetic field is H, respectively.

**Figure 20:**
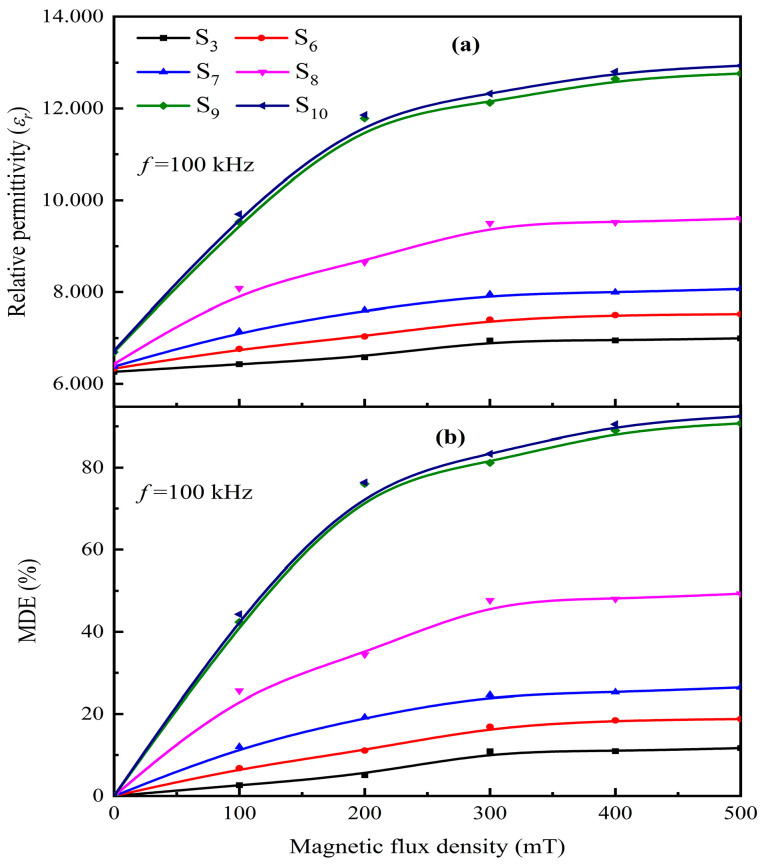
Curves of relative permittivity (**a**) and magnetodielectric effect (**b**) with magnetic field intensity for samples with different particle concentrations.

To further analyze the effect of FG-10 particle concentration on the magnetodielectric properties of microstructurally ordered Fe_3_O_4_@rGO/PDMS-MRE, we tested the relative permittivity and magnetodielectric effect of Fe_3_O_4_@rGO/PDMS prepared by FG-10 at different concentrations under the magnetic fields from 0 to 500 mT at an excitation electric field frequency of 100 kHz and obtained the results as shown in Figure 20. Among them, the FG-10 concentrations were 10 wt%, 20 wt%, 30 wt%, 40 wt%, 50 wt%, and 60 wt%, respectively. It was found from Figure 20a that the relative permittivity of Fe_3_O_4_@rGO/PDMS-MRE increased with the increase of FG-10 concentration at the same magnetic field. The relative permittivity of Fe_3_O_4_@rGO/PDMS-MRE reaches 12.934 when the magnetic flux density is 500 mT, and the filler content is 60%. This is because the increase in the concentration of the composite particles in the MRE leads to an increase in the number of particles per unit volume and a decrease in the particle spacing. According to the Maxwell–Wagner capacitance system model, the increase in the number of particles and the decrease in the particle spacing will increase the total capacitance of the particles in the material itself and the total interparticle capacitance, which manifests as an increase in the relative permittivity of Fe_3_O_4_@rGO/PDMS. Moreover, as the concentration of FG-10 composite particles increases, the particle-to-particle spacing becomes smaller, the interaction force between the particles increases, and mutual polymerization occurs, forming a chain structure and increasing the polarization capacity. When the concentration of FG-10 particles exceeds 50 wt%, the polymerization ability approaches saturation and the amount of particle spacing change decreases, leading to a flattening of the overall trend of relative permittivity increment change of the material.

It can be seen from Figure 20b that the magnetodielectric effect increases with the increase of magnetic flux density and particle concentration, and finally, it basically tends to saturate, which is due to the saturation of FG-10 particles under the magnetic field. When the magnetic flux density is 500 mT and the filler content is 60%, the magnetoelectric effect of Fe_3_O_4_@rGO/PDMS-MRE reaches 92.4%, exhibiting a strong magnetodielectric effect. On the one hand, the strong magnetodielectric effect of the material originates from the combination of the Maxwell–Wagner effect and the magnetoresistance effect [36]. Under the action of the magnetic field, the magnetic properties of the composite system change, and its conductivity also changes, producing a magnetoresistance effect, while the different magnetoresistance responses possessed by the composite particles and the particle boundaries change the charge buildup on both sides of the interfacial layer, which enhances the interfacial polarization and obtains a strong magnetodielectric effect. On the other hand, the strong magnetodielectric effect of the material originates from the magnetostrictive effect. The magnetic field changes the spacing and rearrangement of the directionally aligned flaky Fe_3_O_4_@rGO composite particles so that the material produces strain, generates the magnetostrictive effect and finally obtains a strong magnetodielectric effect.

### 3.4. Mechanistic Analysis of Magnetodielectric Effect of Ordered Microstructured Fe_3_O_4_@rGO/PDMS-MRE

**Figure 21:**
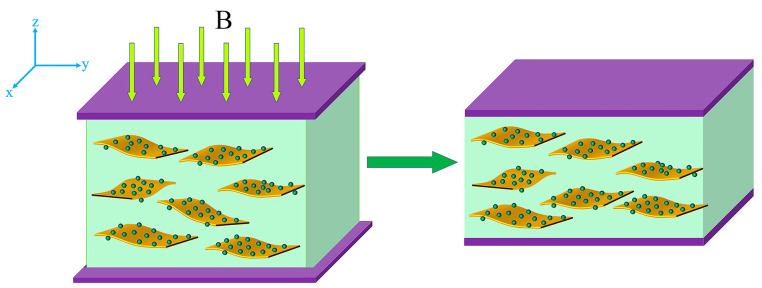
Mechanism diagram of magnetodielectric effect.

The mechanism of the microstructurally ordered Fe_3_O_4_@rGO/PDMS-MRE magnetodielectric effect can be attributed to the following aspects: first, the flaky Fe_3_O_4_@rGO composite particles and the insulating PDMS substrate are distributed in a “sandwich” form, forming a large number of microcapacitors. Figure 21 shows the model of microcapacitor changes before and after the application of the magnetic field. It can be seen from Figure 21 that when the axial magnetic field is applied, the flaky Fe_3_O_4_@rGO composite particles move in the z-axis direction and approach each other, i.e., the axial gap of flaky Fe_3_O_4_@rGO composite particles decreases and the orthogonal area increases under the magnetic field, while the radial gap of flaky Fe_3_O_4_@rGO composite particles increases in the y–z plane, which leads to the number of columns of microcapacitors and the number of series microcapacitors in each column. The number of columns of microcapacitors and the number of series microcapacitors in each column vary with the magnetic flux density. It is the synergistic effect of the reduced distance of the flaky Fe_3_O_4_@rGO composite particles and the rearrangement degree of the composite particles that causes the change in the capacitance value of the microcapacitance, which in turn affects the magnitude of the relative permittivity of the material. In addition, when the Fe_3_O_4_@rGO/PDMS-MRE is placed in an electric field environment, a large number of carriers accumulate between the interfaces of Fe_3_O_4_@rGO nanoparticles and PDMS matrix due to the polarization effect, which also significantly increases the relative permittivity of the material.

## 4. Conclusions

In this paper, five kinds of Fe_3_O_4_@rGO electromagnetic biphasic composite particles were prepared, and the ordered microstructure Fe_3_O_4_@rGO/PDMS-MRE with different Fe_3_O_4_@rGO concentrations was obtained. Their magnetodielectric properties were investigated. The main conclusions were drawn as follows.

(1) Chemical structure and microscopic morphological analysis indicate the successful synthesization of Fe_3_O_4_@rGO nanocomposite particles. It is shown that when the ratio of modified Fe_3_O_4_ to GO is 10:1, Fe_3_O_4_ particles are uniformly loaded on the surface of rGO, and the composite particles have a flaky structure. Moreover, Fe_3_O_4_@rGO composite particles are superparamagnetic at room temperature with a saturation magnetization of 42.36 emu/g, which is beneficial to the preparation of MRE with ordered microstructure.
(2) It is shown that the relative permittivity of Fe_3_O_4_@rGO/PDMS-MRE is sensibly influenced by the magnetic flux density and the filler concentration. In particular, when the magnetic flux density is 500 mT and the filler concentration is 60 wt%, the relative permittivity reaches 12.934 and the magnetodielectric effect reaches 92.4%. It shows a strong magnetodielectric effect.
(3) A mechanism that can describe the magnetodielectric effect of Fe_3_O_4_@rGO/PDMS-MRE is proposed based on the rearrangement degree and the microcapacitance principle of the composite particles. The obtained effect can be used to design new magnetic sensors for detecting environmental parameters.

## Figures and Tables

**Table 1:** The samples of the experimental test.

Sample	Type of Particles	Weight Fraction (wt%)
S_1_	FG-1	10%
S_2_	FG-5	10%
S_3_	FG-10	10%
S_4_	FG-15	10%
S_5_	FG-20	10%
S_6_	FG-10	20%
S_7_	FG-10	30%
S_8_	FG-10	40%
S_9_	FG-10	50%
S_10_	FG-10	60%
S_11_	FG-10 (random)	10%
S_12_	Fe_3_O_4_	10%
S_13_	——	——

**Table 2:** Magnetic field generator structural dimensions.

Parameter	Symbol	Value (mm)
Diameter of upper and lower end covers	*D*_1_	130
Large diameter of coil frame	*D*_2_	110
Small diameter of coil frame	*D*_3_	46
Diameter of disk	*D*_4_	40
Diameter of movable electromagnet core	*D*_5_	40
Length of coil frame	*L*_1_	54
Thickness of MRE	*t*_1_	2
Length of shell	*L*_2_	130
Thickness of shell	*t*_2_	10
Length of disk	*L*_3_	60
Length of movable electromagnet core	*L*_4_	94

**Table 3:** Design parameters of magnetic circuit.

Parameter	Symbol	Value
Relative permeability of S10C steel	$\mu_{D}$	1500
Total magnetomotive force	$F_{m}$	2000 A
Maximum input current	$I_{\max}$	2 A
The coil number of turns	N	1700
Nominal diameter of copper wire	D	0.8 mm
Resistance of copper wire	R	27 Ω

**Table 4:** The magnetic property parameters of Fe_3_O_4_, KH550@Fe_3_O_4_, and FG-10.

Sample	$M_{s}\;(\mathrm{emu}/g)$	$M_{r}\;(\mathrm{emu}/g)$	$H_{c}\;(\mathrm{Oe})$
Fe_3_O_4_	87.24	11.99	0.68
KH550@Fe_3_O_4_	127.52	18.12	1.33
FG-10	42.36	4.28	0.27

## Data Availability

The data presented in this study are available on request from the corresponding author.
